# Microbiota mitochondria disorders as hubs for early age-related macular degeneration

**DOI:** 10.1007/s11357-022-00620-5

**Published:** 2022-08-18

**Authors:** János Fehér, Ágnes Élő, Lilla István, Zoltán Zsolt Nagy, Zsolt Radák, Gianluca Scuderi, Marco Artico, Illés Kovács

**Affiliations:** 1 PRIMAVERA Program, Nutripharma Hungaria Ltd., Budapest, Hungary; 2 Department of Ophthalmology, Semmelweis University, Budapest, Hungary; 3 Research Institute of Sport Science, University of Physical Education, Budapest, Hungary; 4 Ophthalmology Unit, NESMOS Department, Sant’Andrea Hospital, Faculty of Medicine and Psychology, Sapienza University of Rome, Rome, Italy; 5 Department of Sensory Organs, “Sapienza” University of Rome, Roma, Italy; 6 Department of Ophthalmology, Weill Cornell Medical College, New York City, NY USA

**Keywords:** Age-related macular degeneration, Microbiota, Mitochondria, Ferroptosis, Mitochondria contact sites, Lipid droplets, Innate immunity, Microglia, Photoreceptor, Retinal pigment epithelium, Bruch’s membrane, Choriocapillaris, Electron microscopy, Morphometry

## Abstract

Age-related macular degeneration (AMD) is a progressive neurodegenerative disease affecting the central area (macula lutea) of the retina. Research on the pathogenic mechanism of AMD showed complex cellular contribution governed by such risk factors as aging, genetic predisposition, diet, and lifestyle. Recent studies suggested that microbiota is a transducer and a modifier of risk factors for neurodegenerative diseases, and mitochondria may be one of the intracellular targets of microbial signaling molecules. This review explores studies supporting a new concept on the contribution of microbiota—mitochondria disorders to AMD. We discuss metabolic, vascular, immune, and neuronal mechanism in AMD as well as key alterations of photoreceptor cells, retinal pigment epithelium (RPE), Bruch’s membrane, choriocapillaris endothelial, immune, and neuronal cells. Special attention was paid to alterations of mitochondria contact sites (MCSs), an organelle network of mitochondria, endoplasmic reticulum, lipid droplets (LDs), and peroxisomes being documented based on our own electron microscopic findings from surgically removed human eyes. Morphometry of Bruch’s membrane lipids and proteoglycans has also been performed in early AMD and aged controls. Microbial metabolites (short-chain fatty acids, polyphenols, and secondary bile acids) and microbial compounds (lipopolysaccharide, peptidoglycan, and bacterial DNA)—now called postbiotics—in addition to local effects on resident microbiota and mucous membrane, regulate systemic metabolic, vascular, immune, and neuronal mechanisms in normal conditions and in various common diseases. We also discuss their antioxidant, anti-inflammatory, and metabolic effects as well as experimental and clinical observations on regulating the main processes of photoreceptor renewal, mitophagy, and autophagy in early AMD. These findings support an emerging concept that microbiota-mitochondria disorders may be a crucial pathogenic mechanism of early AMD; and similarly, to other age-related neurodegenerative diseases, new treatment approaches should be targeted at these disorders.

**Supplementary Information:**

The online version contains supplementary material available at 10.1007/s11357-022-00620-5.

## Introduction

AMD is a chronic, progressive, and sight-threatening disease of the macula lutea—the small, central area of the retina. AMD affects a large segment of the population, being particularly common among people aged 40 and older. Recent studies estimated the prevalence of AMD to be 8.7% globally, 12.3% in Europe, 8.4% in the USA, and 7.4% in Asia. The estimated global number of people with AMD was 196 million in 2020, with a projecting increase to 288 million in 2040 [1]. In Europe, approximately 67 million people currently suffer from AMD and, due to population aging, this number is expected to increase by 15%, up to 77 million by 2050 [2].

From a clinical and pathologic point of view, atrophic (dry) and exudative (wet) forms of AMD can be distinguished [3]. Early dry AMD is characterized by the presence of small to medium-sized (≥63 to 125 μm) yellowish deposits (drusen) under the retinal pigment epithelium (RPE). Visual acuity is still good, but difficulties in adapting to light or dark may occur, and the center of the visual field becomes fuzzy or shadowed—lines appearing wavy, blurred, or distorted. In intermediate AMD pigmentary abnormalities with medium-sized and at least one large drusen (>125 μm) can be seen in the macula, visual acuity significantly decreases due to blurred central vision for the objects both close by and far away, and a gray spot may develop in the center of the visual field. In late AMD, serious damage to photoreceptors, RPE cells, and choriocapillaris appear, forming “geographic” atrophy in the central area of the retina; and as the disease progresses, with the gray central spot becoming black, people have difficulty in distinguishing colors, details, and later in reading, driving, and working. In approximately 10–15% of cases, the atrophic form may become exudative, characterized by subretinal edema and hemorrhages derived from newly formed vessels of choroidal and, in part, retinal origin (neovascular AMD). This “stroke-like” destruction of the outer retina may occur at any stage of the disease and cause sudden deterioration of visual function [4, 5]. AMD is a bilateral disease, but the progression and severity may be significantly different between eyes; moreover, the atrophic form may occur in one eye, with exudative in the contralateral eye.

Multiple risk factors are involved in the pathogenic mechanism of AMD such as aging, genetic disposition, diet, and lifestyle. Epidemiologic studies from various countries and regions have explicitly shown that age is the main risk factor for AMD, as it affects the senior population with increasing prevalence with aging [6]. Genetic predisposition may contribute to the development and progression of AMD in most patients [7, 8]. Large population-based studies have established genetic dysregulation contributes 46–71% to the expression of the disease, with *CFH* and *ARMS2/HTRA1* being the two most notable risk loci among the 103 identified AMD-associated loci so far [9–11]. Further studies showed that mitochondrial DNA (mtDNA) damage in RPE cells can be an essential element in AMD pathogenesis [12–15]. Finally, other studies demonstrated that the apolipoprotein E (APOE) gene polymorphism is strongly associated with AMD susceptibility [14, 15]. Diet rich in green vegetables, fish, and omega-3-rich oils is beneficial for patients with AMD, as it might delay disease progression and help retain better visual function [16]. The Mediterranean diet, for example, was associated with a 41% reduced risk of incident advanced AMD [17]. Lifestyle and physical activity, in particular, might reduce the risk of early AMD, lowering by as much as 3-fold the risk of advanced AMD in a person’s lifetime—besides the social and economic costs of AMD to society [18, 19]. In contrast, an unfavorable lifestyle (having a western diet, sedentary life, smoking, and some medicines) increased the risk of late AMD at least 2-fold [20]. Current conceptualizations suggests that these risk factors contribute to the development of AMD through metabolic [21–23], vascular [24, 25], immune [23, 26], and neuronal mechanisms [27, 28]. However, the interplay between risk factors and these mechanisms is still not fully explored.

Recent observations support evidence that microbiota is a transducer of environmental signals, modifying the risk of diseases across all ages and that this changes with age. As such, resetting the microbiome-derived signals of “unhealthy” aging through microbiota-associated interventions may be a new approach to preventing and treating age-related diseases [29]. There is a consistent body of evidence that suggests correlations between altered microbiota and the development of neurodegenerative diseases, as low-grade (subclinical) inflammation evoked by microbiota-gut dysbiosis has been described in Alzheimer’s disease [30], Parkinson’s disease [31], and several ocular diseases including AMD [32–35]. Clinical studies also found the different microbial compositions of pharyngeal as well as both oral and nasal samples of AMD cases and controls [36, 37]. Others showed an association between AMD and periodontal diseases such as periodontitis, fewer teeth, and more alveolar bone loss than those without AMD [38]. These observations provide further evidence for the existence of microbial triggers for AMD, but the exact mechanism is not fully explored.

Although there have been major breakthroughs in the treatment of exudative AMD, no efficacious treatment is yet available to prevent irreversible loss of the central vision, and nowadays AMD represents a growing social, medical, and economic burden for industrialized societies [34]. The aims of our paper are: (i) to highlight an emerging concept on the role of microbiota-mitochondria disorders in early AMD supported by our own observations on photoreceptor-RPE, Bruch’s membrane, and choriocapillaris alterations; and (ii) to strengthen microbial treatment approaches recommended before irreversible damage emerges in this common eye disease. Our findings come from a database of surgically removed human eyes collected at the 1st Department of Ophthalmology, Semmelweis University of Budapest, Hungary over the period between 1970 and 1986 when this surgical intervention afforded the best available opportunity to treat certain eye diseases. These specimens were fixed immediately after enucleation; thus, post-mortem changes of the retina rich in unsaturated lipids were prevented. Finally, for the study of membrane-lipids and proteoglycans, we used highly specific and sensitive methods that allowed morphometric analysis and comparison of AMD and aging, as described in detail in the chapter on Material and Methods (see Supporting material).

## Photoreceptor-RPE cells in early AMD

### Mitochondrial alterations

Photoreceptor-RPE cells have high energy demands and a high density of mitochondria that produce ATP through oxidative phosphorylation (OXPHOS) of fuel substrates. Similar to other tissues with high metabolic rates, photoreceptors metabolize fatty acids through OXPHOS [35]. Photoreceptors are prone to photo-oxidative damage and approximately 10% of the scheduled apical photoreceptor outer segment (POS) membranes are daily engulfed by the RPE, which are specialized phagocytes responsible for the turnover of POS in normal conditions [39]. However, in vitro studies have shown a moderate decline of RPE phagocytosis with age, though this was significantly reduced in AMD as compared to age-matched controls, highlighting the importance of RPE phagocytic dysfunction in AMD [40]. Other in vitro studies demonstrated that cultured human RPE from AMD donors were functionally impaired. Moreover, they showed reduced mitochondrial activity associated with increased susceptibility to oxidative stress, higher levels of ROS under stress conditions, and impaired autophagy as compared to RPE from normal donors. These studies proposed that impaired phagocytosis and mitochondrial activity—as well as dysfunctional autophagy in RPE and accumulation of metabolic by-products—may be underlying mechanisms contributing to the pathophysiology of AMD [41].

Additionally, electron microscopy of cultured human RPE cells showed marked differences in the structural features of the mitochondria with aging. RPE cells from young and middle-aged donors have numerous mitochondria with regular-sized, round, or oval-shaped, distinctly visible cristae, and the outer membrane appeared intact. RPE cells from aged donors contained mitochondria sparsely distributed in the cytoplasm, with an irregular size, electron-dense matrix, less distinct cristae, and disrupted outer membrane [42]. These mitochondrial alterations correspond to those that are characteristic of iron overload observed in other organs [43]. Further studies showed that increased ferrous ion (Fe2+), and subsequent generation of hydrogen peroxide and hydroxyl free radicals formed within the inner mitochondrial membrane, may cause severe mitochondrial dysfunction and subsequent photoreceptor death, the so-called ferroptosis [44].

Ferroptosis—iron-dependent cell death—is, from a biochemical and morphological point of view, different from other forms of regulated cell death such as apoptosis, necrosis, and pyroptosis [45]. Ferroptosis contributes to numerous severe and common diseases in humans, such as stroke, traumatic brain injury, ischemia-reperfusion injury, non-alcoholic steatohepatitis, cardiomyopathy, metabolic syndrome, and neurodegenerative diseases [46, 47]. Increased iron levels have been reported in the RPE, outer retina, and choroid in the elderly [48]. Previous studies suggested that ferroptosis may occur also in AMD, as phagocytosis of POS discs rich in iron may promote iron accumulation in the RPE. This accumulated iron either binds to melanin in RPE cells or accumulates to toxic levels, and further studies confirmed that retinal iron accumulation is a characteristic feature of AMD [49, 50]. Excessive ferrous ion (Fe2+) subsequently generates RO via Fenton reactions, and ROS further reacts with PUFAs, promoting lipid peroxidation, which causes mitochondrial destruction and subsequent death of RPE and photoreceptors by ferroptosis [51, 52]. Furthermore, mitochondrial ferritin, an iron-sequestering protein, is expressed in cell types characterized by high metabolic activity and oxygen consumption, including the human retina [53].

Previous electron microscopic studies from our laboratory showed a decrease in the number and area of mitochondria as well as loss of mitochondrial cristae and matrix in RPE cells—this being significantly more severe in early AMD as compared to age-matched controls—which we considered to be signs of apoptosis [54]. In a reappraisal of these images—among mitochondria that appeared to be normal and damaged—we also found mitochondria characterized by their smaller than normal volume and condensed mitochondrial membrane densities, as well as by diminished or vanished cristae, rupture of their outer membrane, and electrodense inclusions in both aged eyes and early AMD. These ultrastructural features of mitochondria are similar to those seen in iron overload; as such our findings may support the role of ferroptosis in early AMD. However, further studies are certainly needed to detect quantitative differences between AMD and aging.

Since RPE metabolism supports the light-induced retinoid cycle [55, 56], one of the possible consequences of mitochondrial dysfunction in early AMD may manifest as a disorder of light-induced retinoid recycle. In fact, impaired dark adaptation was found in AMD [57–60]; as well as decreased sensitivity [61] and increased recovery time after photostress was found in early AMD [62, 63], suggesting impaired energy production in this disease.

**Fig. 1:**
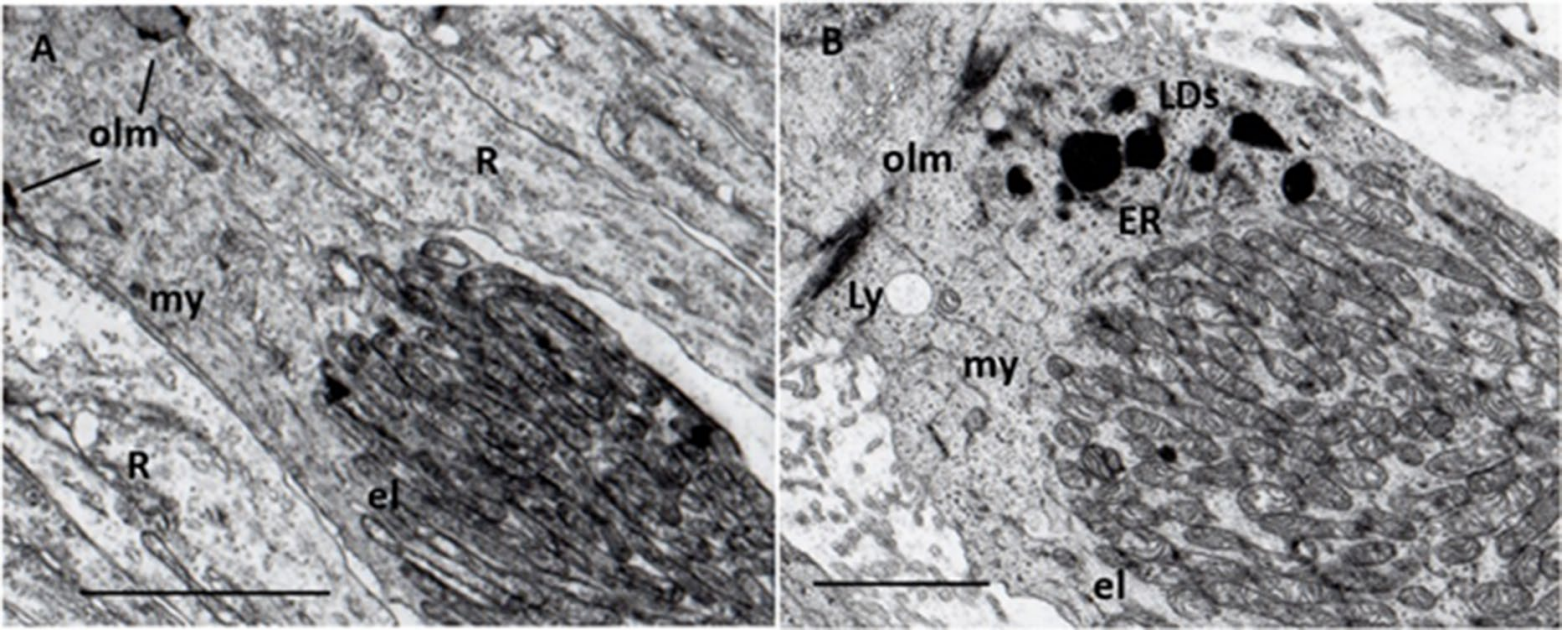
Photoreceptor inner segment. **A** Normal aged cone and rods. Ellipsoid (el) contains numerous, densely packet, uniformly long mitochondria with well-preserved cristae and matrix. Myoid (my) contains a few endoplasmic reticula (ER) and normal cytoplasm free from inclusions, similarly to neighboring rods (R). olm: outer limiting membrane; 76 years; male; ×12,000; bar: 1 μm. **B** Early AMD cone. Ellipsoid (el) contains mitochondria of various shapes and sizes with focal loss of cristae and matrix. Myoid (my) is shorter and contains some endoplasmic reticulum (ER), lysosome (Ly), electrodense, and lipid-containing inclusions (LDs) next to the mitochondria. olm: outer limiting membrane; 62 years; male; ×20,000; bar: 0.1 μm

Additionally, mitochondria of photoreceptors’ inner segment are affected in early AMD. Optical coherence tomographic (OCT) studies reported that the average relative ellipsoid zone intensities (distal part of the inner segment) were significantly reduced in eyes with intermediate AMD compared to normal eyes and with increasing age [64, 65]. Furthermore, OCT and histopathologic studies suggested, that mitochondrial alterations may be responsible for changes in ellipsoid zone reflectivity that may indicate early photoreceptor damage [59]. Animal studies showed that oxidative stress in RPE leads to mitochondrial dysfunction in both RPE and photoreceptors [22]. Our electron microscopy of aged human retinas confirmed these mitochondrial alterations in ellipsoid; what is more, various forms of electrodense (possible lipid-containing) granules were observed in the myoid in early AMD (Fig. 1A,B).

### Mitochondria contact sites in the RPE cells

**Fig. 2:**
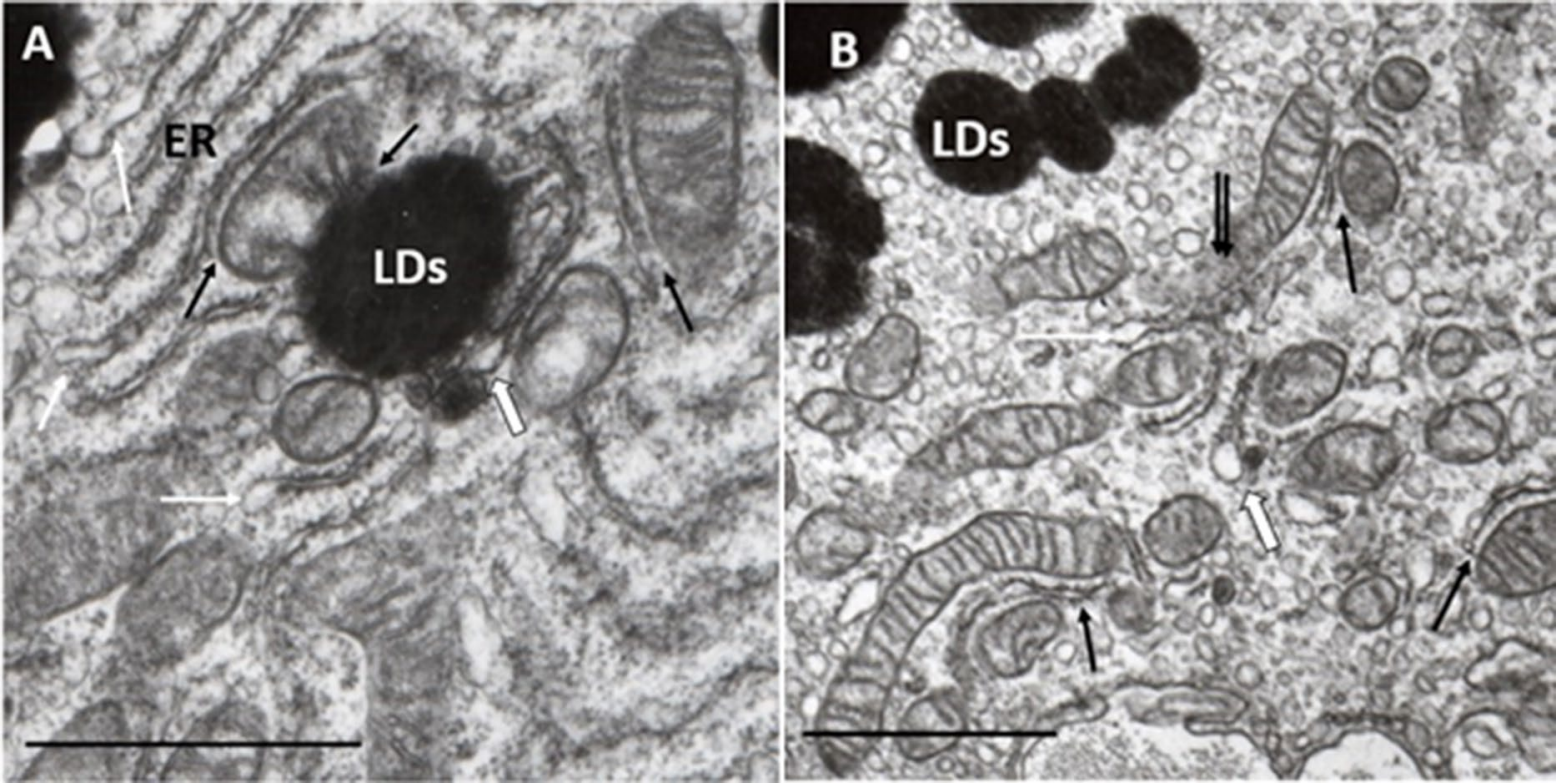
MCSs in the RPE. **A** MCSs in young RPE (normal). Typical organization of contact sites between mitochondria, endoplasmic reticulum (ER), and LDs (black arrows). ER show dilatation at some places, phenomena of ER-stress (white arrows), one of these dilatations is continuous with a small LD (widened white arrow). Eight years; male; ×32,000; 1 μm. **B** MCSs in adult RPE (normal): Several contact sites between mitochondria and ER (black arrows), ER show dilatation at some places, phenomena of ER-stress (white arrows), one of these dilatations is continuous with a small LD (widened white arrow). At the center of the pictures, multiple contact sites and presumed mitophagy can be seen (double black arrows). Thirty-eight years; male; ×24,000; 1 μm

MCSs or synonymously mitochondria-associated membrane (MAM) are recently identified, but well studied, special subcellular structural and functional networks of organelles, such as mitochondria, and the endoplasmic reticulum (ER), LDs, lysosomes, and peroxisomes. MCSs are characterized by specific structural and functional properties at the sites of closely apposed membranes, and proteins tethering two organelles have also been defined [60]. MCSs are dynamic structures, and these contact sites serve as unique intracellular platforms regulating a wide range of cellular reactions, including autophagy through non-vesicle contact [66]. This extensive communication through membrane contact sites—including disturbing inter-organelle communication—is intimately linked with age-related pathologies [67]. (Fig. 2A,B)

MCSs formed between mitochondria and ER have been the most widely studied, with it being proposed that they play crucial roles in various biochemical and signaling functions such as Ca2+ homeostasis, ROS production, lipid biosynthesis, and trafficking—alongside the regulation of mitochondrial division, fusion, and mtDNA replication, autophagosome biogenesis, and autophagy [68]. These contacts are also involved in different cell dysfunctions and pathologies, including gastrointestinal inflammations, and colorectal cancer [69], traumatic brain injury [70], neurodegenerative diseases [71], metabolic diseases [72], cardiovascular diseases [73], and mood disorders [74], as well as with aging and age-related diseases [75, 76].

LDs are particular forms of cytoplasmic lipid storage that are now considered to be organelles composed of a neutral lipid core, comprising triacylglycerols and sterol esters, surrounded by a phospholipid monolayer with several decorating proteins, called perilipins [77, 78]. Perilipins are conserved proteins and are essential for lipid metabolism [79]. LDs originate from the ER, with which they maintain close contact throughout their life cycle, and their junctions facilitate the exchange of both lipids and proteins between these two compartments. LDs also form contact sites with ER-derived catabolic organelles, such as autophagosomes, lysosomes, autolysosomes, and late endosomes. These contacts support lipid catabolism through lipophagy or lipolysis and supply lipids for autophagosome biogenesis [80]. LDs have become one of the most exciting areas due to the important part they play in energy homeostasis, as they seem to be the principal regulators of cellular metabolism. They are also essential components of cellular stress responses, as LDs in stressed cells maintain energy and redox homeostasis and protect against lipotoxicity by sequestering toxic lipids (lipofuscin and other metabolic by-products) into their neutral lipid core. Their mobility and dynamic interactions with mitochondria enable the efficient delivery of fatty acids for optimal energy production. Furthermore, LDs contribute to the removal of protein aggregates when severe proteotoxic stress overwhelms the proteasomal system [81].

LDs form contact sites with mitochondria for metabolic channeling of the released fatty acids toward beta-oxidation [82]. Contacts not only supply lipids for β-oxidation in mitochondria and peroxisomes but may also transfer toxic lipids—as well as misfolded and harmful proteins—to LDs. Mitochondrial function has been shown to be the main determinant of functional lipid storage and oxidation, and adequate regulation of cellular lipid storage and oxidation is indispensable for the maintenance of cellular energy homeostasis and health. Recent studies have identified a subpopulation of mitochondria attached to LDs—denominated peridroplet mitochondria—that have distinct bioenergetics, proteome, cristae organization, and dynamics that support LD build-up as compared to cytoplasmic mitochondria [83, 84]. LDs also contribute to innate immunity as they organize and use immune proteins to kill intracellular pathogens as well as being central players in the local and systemic metabolic adaptation to infection [85]. Finally, they also maintain a symbiotic relationship with autophagy and act as reservoirs of bioactive lipids that regulate innate immunity [86, 87]. Increasing evidence suggests significant roles for LDs in metabolic disease and inflammation, in which dysregulation of lipid metabolism was found to be associated with neurodegeneration, as in Alzheimer’s and Parkinson’s disease [88, 89]. Most recent studies found that accumulation of LDs promotes RPE dysfunction in vitro [90].

Lysosomes play a crucial role in mitophagy, one of the key processes in AMD [14, 91, 92]. Mitochondria are constantly in a dynamic and regulated balance of fusion and fission processes that is known as mitochondrial quality control (MQC). Mitophagy, a major mechanism of MQC, can selectively degrade dysfunctional mitochondria and maintain mitochondrial integrity and function, preventing release of damaged mitochondria-derived ROS. Mitochondrial dysfunction and failing MQC are major determinants of aging and age-related neurodegenerative diseases. MCSs are an intimate topographic and functional relationship between mitochondria and lysosomes that represents the fact that lysosomal degradation of dysfunctional mitochondria is the final step of mitophagy [93–95]. Recent studies have demonstrated that activated mitophagy will prevent damaged mitochondria-derived ROS triggering oxidative stress and inflammatory response, which are the leading causes of metabolic diseases [96].

Daily phagocytosis of shed POS leads to the accumulation of non-degradable inclusion bodies, called lipofuscin (“age pigment”), within lysosomes of the RPE. Detection of autofluorescence from lipofuscin is a widely used diagnostic tool in retinal diseases. Lipofuscin content is lower at the fovea but increases with eccentricity and age, in contrast to those of melanolipofuscin, which is equally distributed at the foveal and perifoveal locations with no age-related changes. These differences suggest different geneses, i.e., cone or rod origin [97].

Peroxisomes are multifunctional, dynamic, and membrane-bound organelles with important functions in fatty acid β-oxidation and ROS metabolism, in the oxidation of very long-chain fatty acids (VLCFAs), and in the biosynthesis of phospholipids. Peroxisomes communicate with other subcellular organelles, such as the mitochondria, ER, LDs, and lysosomes through contact sites [98, 99]. Peroxisomal activities decline with age, and several studies have suggested that peroxisomal dysfunctions might be associated with the pathogenesis of age-related neurodegenerative diseases [100]. Peroxisome proliferator-activated receptors (PPARs) are a family of nuclear receptors that play an essential role in modulating cell differentiation, inflammation, and metabolism. Studies suggested that PPARs may be a therapeutic target for MQC, autophagy, and antioxidant activity—and in treating AMD, dry eye, and diabetic retinopathy [101, 102].

**Fig. 3:**
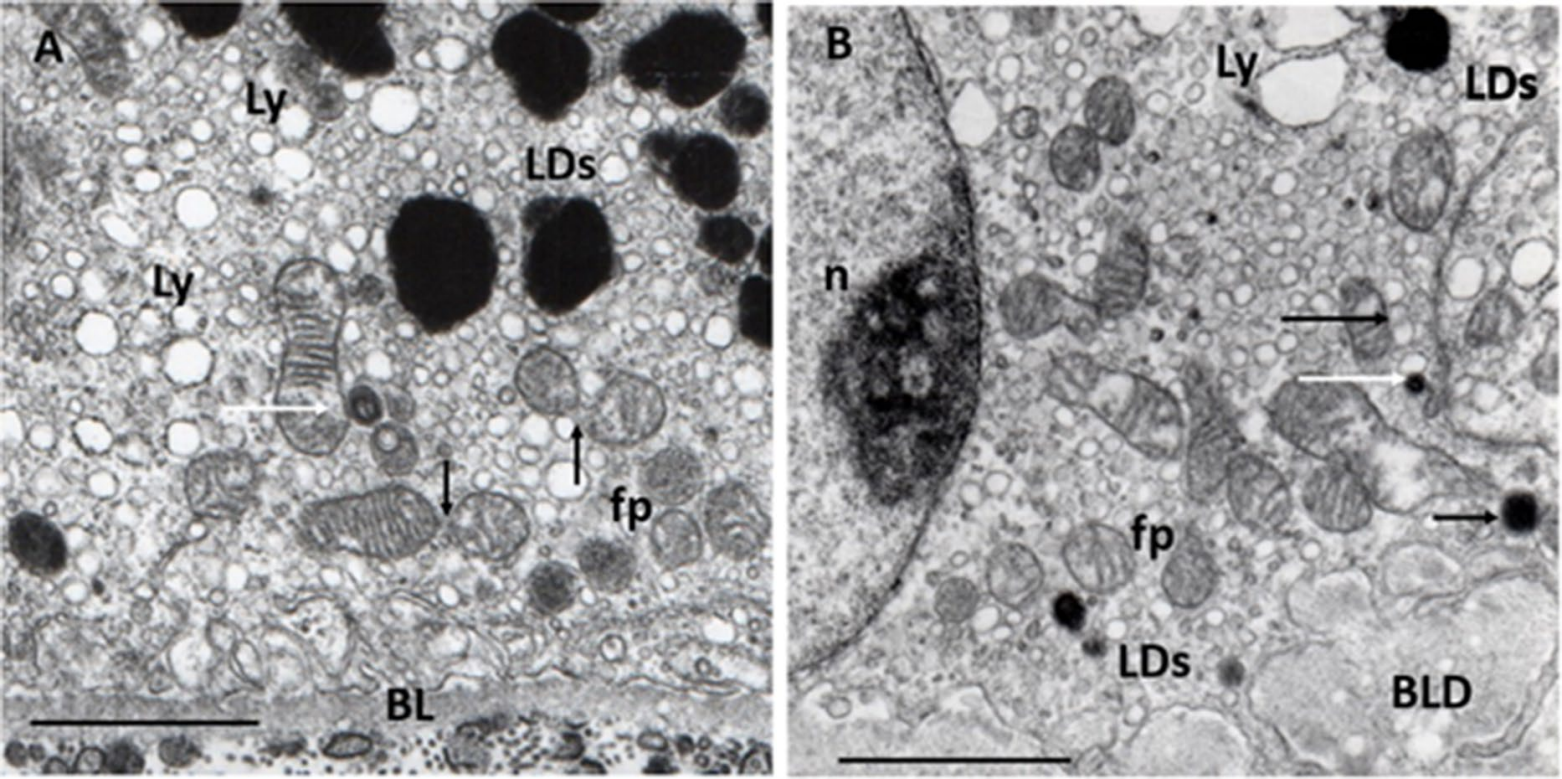
RPE alterations. **A** Normal aged RPE. Characteristic ultrastructural features: sparsely distributed mitochondria in the cytoplasm, which contains numerous small vacuoles features of the lysosome (Ly) and electrodense LDs of various sizes (LDs); mitochondrial cristae and matrix mostly with normal appearance, one of them forms a contact site with small peroxisome (white arrow); two of them show mitochondrial fission (black arrows); some small round-form mitochondria with confluent electron-dense cristae characteristic for iron overload seen in ferroptosis (fp); basal lamina (BL) of RPE shows normal appearance. Eighty-one years; female; ×22,000;1 μm. **B** Early AMD. Characteristic ultrastructural features: a few sparsely distributed mitochondria in the cytoplasm which contains numerous (Ly); focal loss of mitochondrial cristae and matrix, one of them forms a MCSs with LDs and lysosome (black arrows) and one contact site between a small LDs and lysosome (white arrow); some small round-form mitochondria with confluent electron-dense cristae suggesting seen in ferroptosis (fp); LDs in the cytoplasm (LDs) one in the upper right angel, some smaller in the basal cytoplasm of RPE; BLD with some round-form electron-translucent areas characteristic to non-membrane-bound cholesterol. *n*: nucleus. Seventy-two years; male; ×22,000; bar: 1 μm

We have identified for the first time MCSs in human RPE cells from young, aged-normal, and early AMD eyes. MCSs seems to decrease with the age-related decrease of mitochondria, but further studies are needed to quantify these preliminary observations, in particular their relation to AMD (Fig. 3A,B).

### Mitochondria targeted treatment

Supplements containing mitochondriotropic (mitotropic) compounds (L-carnitine, CoQ10, and DHA/EPA) arrested the progression of both visual impairment and fundus alteration in early AMD—as shown by a randomized, placebo-controlled, double-blind clinical trial with long-term follow-up observations [103]. L-carnitine is a lipid transporter from the cytoplasm into mitochondrial β-oxidation, thus it enhances the production of energy from lipids, whilst contributing to the clearance of lipids derived from photoreceptor turnover. Coenzyme Q10 is a component of the electron transport chain and also plays a part in the plasma membrane redox system. While it is produced endogenously, aging and widely used cholesterol-lowering statins compromise the CoQ10 synthesis. DHA and EPA are essential omega-3 fatty acids that are critical components in mitochondrial membrane structure and function. The synergistic effects of these mitochondrial compounds may prevent the generation of ROS in contrast to antioxidant vitamins, which may have benefits for neutralizing ROS. This supplement is available on the market in some EU countries.

Elamipretide (SS-31), a small mitochondrially targeted synthetic molecule, exhibits therapeutic effects and safety in several mitochondria-related diseases [104]. Elamipretide exerts its protective effects by binding to cardiolipin (CL), a mitochondria-specific lipoprotein, vital to normal mitochondrial structure and functions. CL is exclusively localized to mitochondrial membranes in mammalian mitochondria where it stabilizes the inner membrane proteins that are closely linked with electron transport and OXPHOS. CL contains two phosphatidic acid groups linked by glycerol and four linoleic acid groups (C18:2), which are essential for normal CL functions. Elamipretide, this water-soluble tetrapeptide, has free radical scavenging properties. It exerts antioxidant and anti-apoptotic effects in both in vitro and in vivo models of neurodegeneration, ischemia–reperfusion injury, cardiac diseases, and insulin resistance. Extensive studies have shown that where elamipretide is bound to CL it enhanced mitochondrial respiration, activated neural mitochondrial biogenesis, enhanced mitochondrial fusion, inhibited mitochondrial fission—as well as increased mitophagy and retinal function. Currently, elamipretide is an ongoing phase II clinical trial in intermediate AMD [105].

## Bruch’s membrane in early AMD

### Soft drusen LDs

Soft drusen, a clinical and histological hallmark of AMD, may appear as well-defined single or multiple focal deposits of metabolic by-products localized between the RPE basal lamina and the inner collagenous layer of Bruch's membrane [106–108]. In an electron microscope, electron-dense material indicates unsaturated lipids of photoreceptor origin, while poorly electron-dense (“empty”) vacuoles are an accumulation of cholesterols and cholesterol esters that may be a sign of unneeded lipids from the choriocapillaris [24, 109, 110]. The accumulation of numerous or confluent drusen, especially in the macula, is a significant risk factor for the development of advanced AMD. Clinical studies suggest that some visual functions (visual acuity, as well as mesopic and dark-adapted microperimetry) might indicate structural changes related to drusen volume in early AMD [111].

**Fig. 4:**
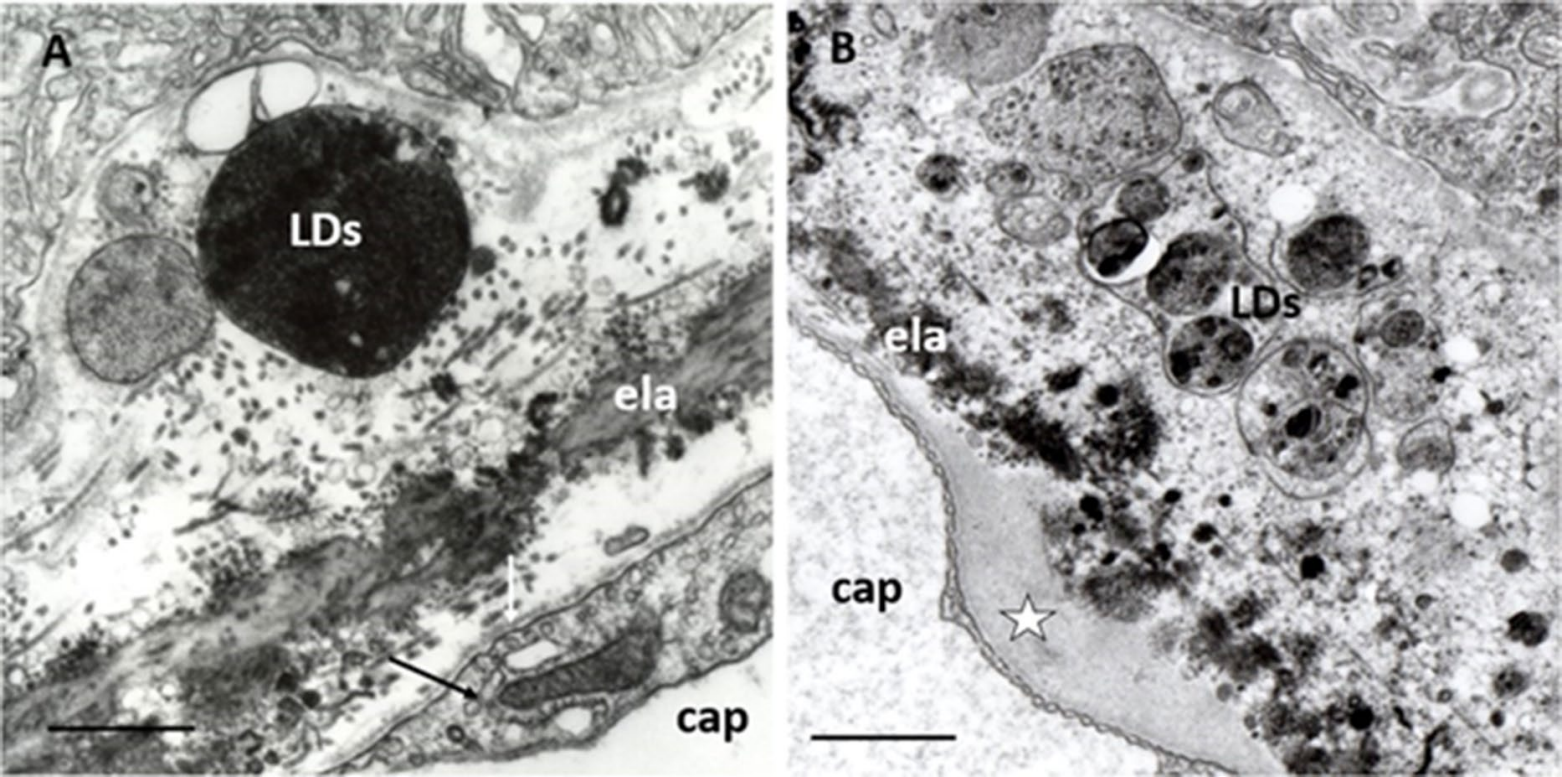
Drusen in early AMD. **A** LDs in early AMD. LDs of different electron densities and sizes are located under the well-circumscribed elevation of the RPE basal lamina (LDs). All other layers of BM show normal aged features. Note a MCS with ER in the endothelial cell (arrow) and several pinocytotic or exocytotic vesicles next to the BM (white arrow). Seventy-one years; female; ×22,000; bar 1 μm. **B** Drusen in early AMD. Electron microscopy of drusen containing LDs of various sizes and electron-density, membrane-fragments, and electron-translucent vacuoles surrounded by appearance homogeneous material. Endothelial cell shows normal fenestration and a dome-shaped basal laminar thickening (star). Eighty-one years; female; ×22,000; bar: 1 μm

We observed, in addition to these histologic features, LDs between basal lamina of RPE and the inner collagenous layer of Bruch’s membrane, suggesting that LDs may occur extracellularly and contribute to the formation of soft drusen (Fig. 4A,B). The biological functions of extracellular LDs remain an interesting question, specifically their possible role in stimulating vascular, immune, and neural reactions from the choroid in dray AMD—alongside generation neovascularization and exudation/hemorrhage in wet AMD.

### Basal laminar thickening of RPE

**Fig. 5:**
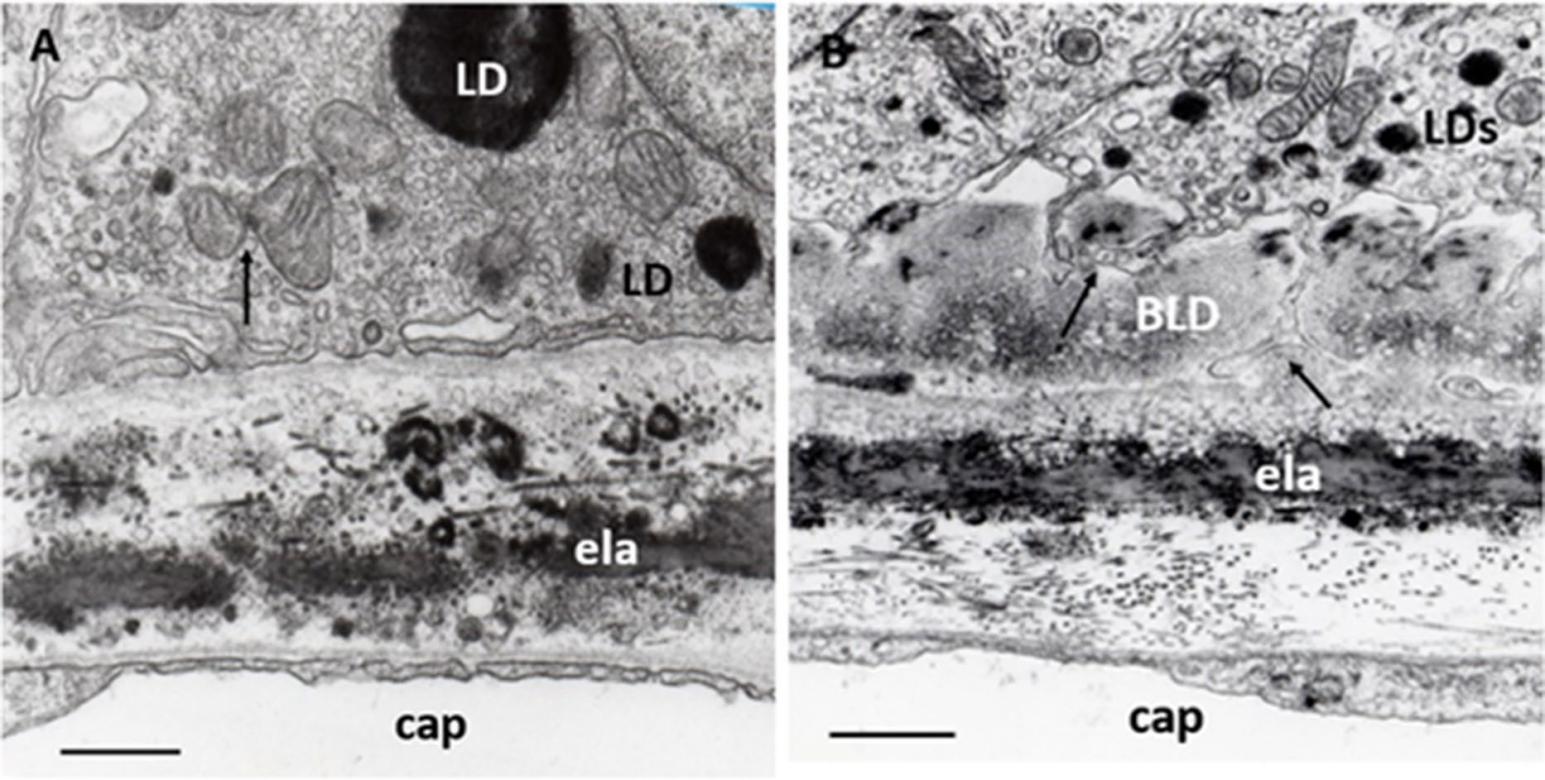
BLD in early AMD. **A** Normal aged Bruch’s membrane. RPE contains some small mitochondria, two of them show fission (arrow) and electrodense LDs; inner and outer collagenous layers contain electrodense inclusions of various shapes and sizes, as well as some of them are round and single membrane-bound; elastic layer**,** basal lamina of RPE, and capillary endothelium are normal. ela: elastic layer, cap: choriocapillaris. Seventy-four years; male; ×28,000; bar: 1 μm. **B** BLD in early AMD. A longitudinal section of BLD between the basal lamina and basal cytoplasm of RPE. In some places, it is amorphous in appearance with small electron-translucent vacuoles, while in other palaces it shows filamentary structures with electron-dense patches due to lipids. Basal in-foldings of the RPE in some places may form deep protrusions into the BLD, reaching the basal lamina of the RPE (black arrows). The cytoplasm of RPE contains numerous small LDs and only a few mitochondria of various sizes but well-preserved cristae and matrix. ela: elastic layer, cap: choriocapillaris. Seventy-one years; female; ×25,000; bar:1 μm

Metabolic disorders of the photoreceptor-RPE in addition to the formation of drusen are considered to be responsible for further alterations of Bruch’s membrane. Lipid accumulation and protein modifications in the Bruch’s membrane are considered further representative phenomena of AMD [112, 113]. The electron microscopic hallmark of AMD is focal or diffuse thickening of the basal lamina of the RPE, called basal laminar deposit (BLD) [114, 115]. We found filamentary structures decreed with electrodense lipids (Fig. 5A,B). Progressive accumulation of lipids (mainly phospholipids) in Bruch's membrane in relation to age was found, and the lipid-rich barrier in Bruch’s membrane may be implicated as a cause of photoreceptor-RPE dysfunction [116]. Cholesterol esters were also identified in Bruch’s membrane which appears as a highly anisotropic “Maltese cross” under a polarization microscope [108]. AMD and atherosclerotic cardiovascular disease may share common pathogenic mechanisms as Bruch's membrane ages—such as arterial intima, for which plasma lipoproteins are the known source of extracellular cholesterol [117]. Both drusen and BLDs are now also detectable by OCT [118].

**Fig. 6:**
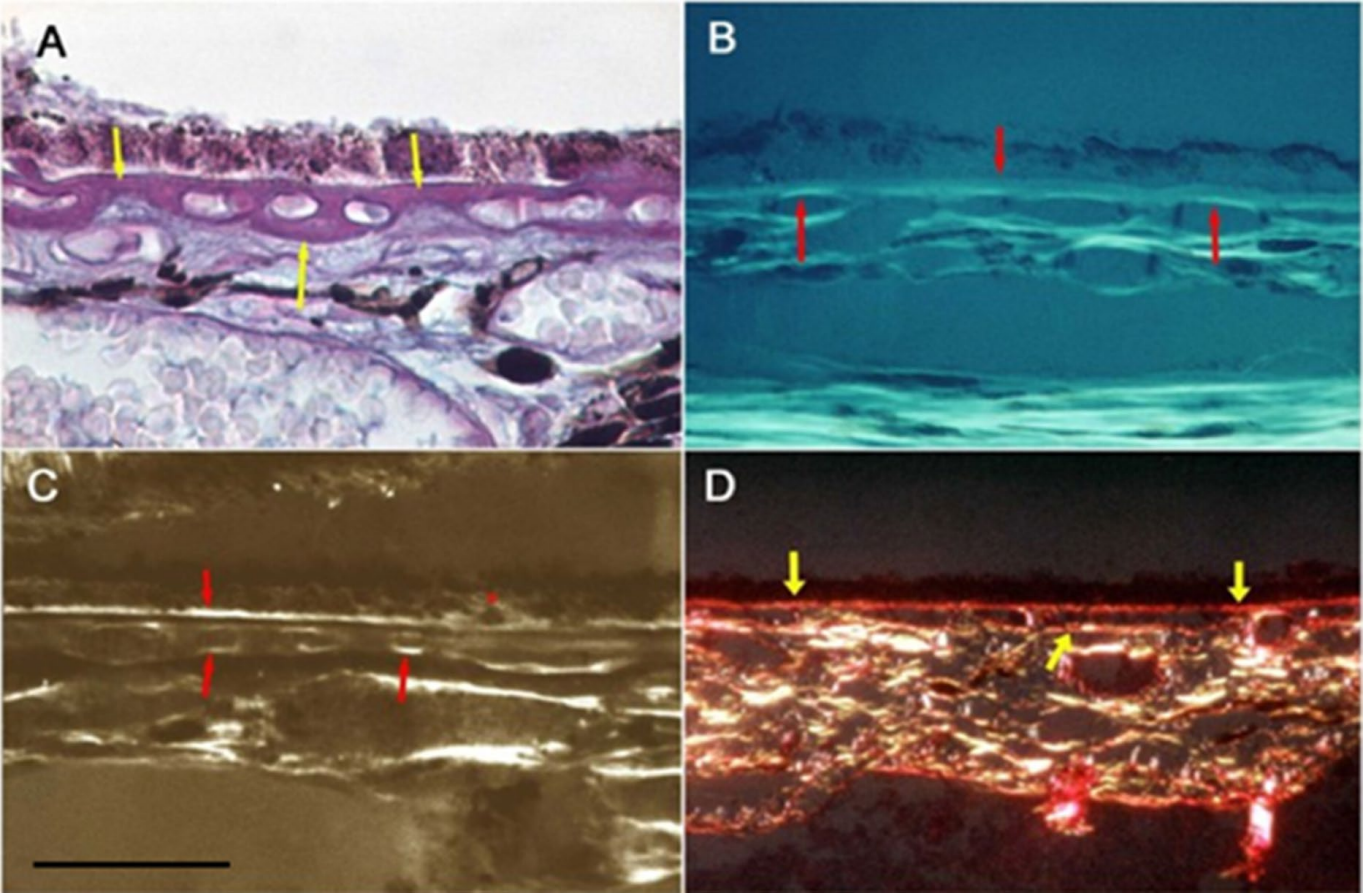
Collagen, lipids, and proteoglycans in Bruch’s membrane of aged normal eyes. **A** Light microscopy showed PAS-positive staining in the Bruch’s membrane (arrows), as well as in the intercapillary and pericapillary connective tissue, indicating their high carbohydrate content**.** PAS alcian-blue hematoxylin staining, 72 years; male; ×350; bar: 10 μm. **B** Collagen fibrils in Bruch’s membrane. After the collagen-specific phenol reaction, two layers of Bruch’s membrane showed intense anisotropy with polarization microscopy. They were prominent just beneath the RPE and much less in the capillary wall (arrows). Seventy-six years; male; ×350; bar: 10 μm. **C** Lipids in the basal lamina of RPE and capillary walls. Polarization microscopy of unstained sections showed anisotropy just beneath the RPE and in the choriocapillaris wall indicating the presence of oriented structures in both layers (arrows). This anisotropy was completely abolished by lipid extraction. The basal lamina of choroidal vascular endothelia also appears bright, while all other structures of the choroid are dark. 82 years; female; ×350; bar: 10 μm. **D** Proteoglycans in the basement membrane of RPE and choriocapillaris. Bruch’s membrane was strongly basophilic and intensively anisotropic after the ABT reaction. This anisotropy was due to the carbohydrate components of the basement membrane of RPE and choriocapillaris as well as those of the inner and outer collagen layers of the Bruch’s membrane (arrows). Seventy-one years; male; ×350; bar: 10 μm

**Fig. 7:**
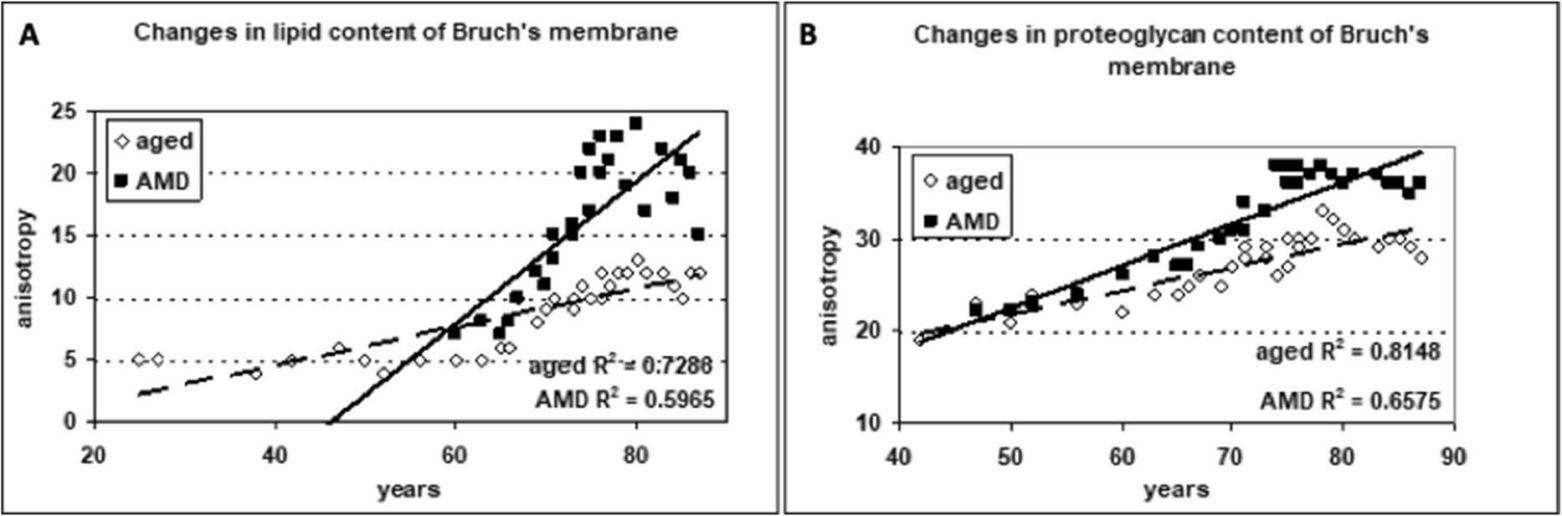
Morphometric analysis of Bruch’s membrane in early AMD as compared to age-matched controls. **A** The lipid content of Bruch’s membrane increased significantly in both aged and AMD groups. The coefficient of correlation for lipids was significantly greater in the AMD group than the age-matched normal controls (*p* < 0.001). **B** The proteoglycan content increased significantly more in AMD compared to normal aging until 75 years (*p* < 0.01)

The use of polarization microscopy—which is a highly sensitive method for detecting lipid, proteoglycan, and collagen structures (but not for amorphous material)—revealed further new details of the age-related changes in Bruch’s membrane, specifically those relating to BLD (Fig. 6A,B,C,D). Histochemical reactions, called “topo-optical reactions”, may further increase the sensitivity and specificity of this microscopic technique. Based on the measurement of anisotropy permitted a semi-quantitative analysis of BLD components and comparison of their changes with aging versus AMD. The anisotropy (“brightness”) of lipids, proteoglycans, and collagen increased with age, but it was significantly higher in early AMD for lipids and proteoglycans (Fig. 7A,B). Our findings confirmed previous observations on the composition of BLD in AMD and added some new aspects of the structural organization and morphometry of lipids and proteoglycans in early AMD as compared to aged controls. Similar age-related thickening and compositional changes were found in retinal capillaries of diabetics and age-matched controls. Furthermore, these alterations were significantly more pronounced in diabetics, as shown in the morphometric analysis [119–121]. The thickening of the capillary wall and accumulation of by-products begin at the Müller glial side, similarly to the Bruch’s membrane, where these alterations appear next to the RPE. These morphological similarities may come from at least partly the common pathogenic mechanism of AMD and diabetic retinopathy.

## Choriocapillaris in early AMD

### Endothelial alterations

Endothelial cells of the choriocapillaris have thin and fenestrated cytoplasm comparable to the glomerular endothelial of the kidney. This ultrastructural feature is considered to indicate an intensive metabolic exchange between blood and the RPE cells. Choriocapillaris microcirculation evidently plays a nutritional role in photoreceptor-RPE metabolism and a well-documented association was found between impaired microcirculation and AMD [122, 123]. With the use of OCTA, recent studies have demonstrated that choriocapillaris flow alterations are closely associated with the development and progression of AMD [124]. Vascular density values of the choriocapillaris—in addition to both superficial and deep retinal capillary plexus—were smaller in early AMD, and the flow-void area in choriocapillaris was greater in early AMD than controls [125]. Patients with early AMD also exhibited signs of systemic and retinal vascular alterations that correlated with known risk markers for future cardiovascular morbidity [126]. Attenuation of the submacular choriocapillaris and endothelial cell loss occurs in early AMD, suggesting a role of altered blood supply in the development of functional and structural abnormalities [127, 128]. Even more, choriocapillaris breakdown already occurs during normal aging and precedes degeneration of the RPE, and loss of endothelial cells of the choriocapillaris is one of the earliest detectable events in AMD [129, 130]. Linear modeling found a statistically significant relationship between dry AMD stage and choriocapillaris perfusion, most prominent in the more peripheral regions of the macula [131]. Average foveal choroidal blood flow in the dry AMD is lower than that of age-matched controls, and the effect is caused mainly by a decrease in volume but not the velocity of blood flow [132]. There is a systematic decrease in choroidal circulatory parameters with an increase in the severity of AMD features associated with risk for the development of CNV, suggesting a role for ischemia in the development of CNV [133]. Further studies confirmed these observations; namely, that decreases in the foveolar choroidal circulation precede the development of CNV in AMD [134], and lower choroidal perfusion is a risk factor for the development of CNV in the fellow eye of patients with unilateral CNV [135].

**Fig. 8:**
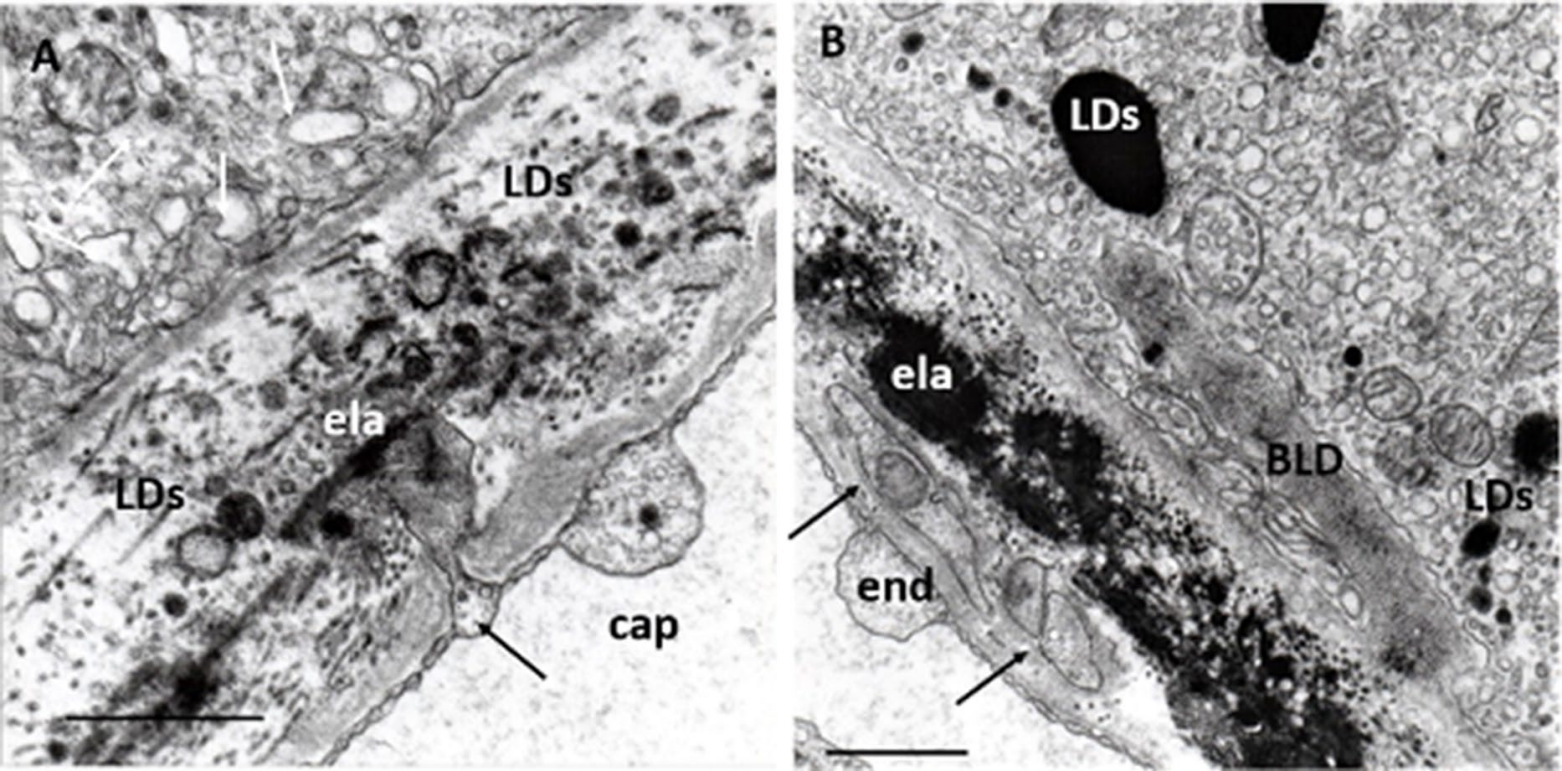
Endothelial alterations. **A** Endothelial sprouting in a normal eye. Small, deep sprouting reaches the elastic layer of the Bruch’s membrane (black arrow), which contains several LDs of various sizes and shapes can be seen. Numerous dilated lysosomes and small round-form lysosomes next to a mitochondrion can be seen in the RPE (white arrows) ela: elastic layer; 48 years; female; ×32,000; bar: 1 μm. **B** Endothelial cell processes of in early AMD. Transversal section of endothelial cell processes in the basal lamina of the choriocapillaris (arrows). They contain small mitochondrion with confluent cristae. BLD, some small sparsely distributed mitochondria, several lysosomes, and LDs can be seen in the RPE. ela: elastic layer. Eighty-four years; female; ×22,000; bar: 1 μm

Early transmission and scanning electron microscopy have shown that choriocapillaris endothelial cells in some places may undergo the loss of fenestration and form sprouting or pseudopodia-like cytoplasmic processes invading the Bruch’s membrane [136]. Our observations confirmed these findings: that sprouting oriented toward the RPE rarely projected from the lateral side of the choriocapillaris, suggesting a functional relationship between the RPE and endothelial cells. In most cases, the loss of fenestration was accompanied by the thickening of both cytoplasm of endothelial cells and the basement membrane of choriocapillaris next to the sprouting (Fig. 8A). Cytoplasmic processes of unknown origin were found in the thickened basal lamina of choriocapillaris. The origin of these cell processes can hardly be verified based on only electron microscopy. Theoretically, they may come from invading endothelial cells, fibrocytes of the choroid, or blood-derived mononuclear cells (Fig. 8B).

Choriocapillaris, in addition to their role in blood supply, are also involved in physiologic innate immune processes, and disorders of choriocapillaris through the innate immune system and local inflammation may contribute to the pathophysiology of AMD [137]. Vascular endothelial cells—similarly to macrophages—have several innate immune functions including phagocytosis, cytokine secretion, and antigen presentation, sensing both pathogen- and damage-associated molecular patterns, as well as proinflammatory, immune-enhancing, anti-inflammatory, immunosuppression, migration, and plasticity. Based on these observations, endothelial cells are now considered to be innate immune cells that have immune tolerogenic, memory, and immuno-metabolic functions not only in infections but also in metabolic diseases [138]. Thus, we may speculate that the formation of cytoplasmic sprouting and processes are seen under the electron microscope may indicate innate immune functions.

### Immune cell alterations

Emerging evidence supports the concept that AMD is at least in part an inflammatory disease, as the accumulation of metabolic byproducts evokes inflammatory reactions as signals of impaired innate immunity [139–141]. Macrophage or microglia is resident immune cells of the human choroid, and in physiological conditions, they perform a housekeeping role in the complement-mediated phagocytosis and removal cell-debris and metabolic byproducts of the photoreceptor-RPE cell turnover. Complement activation products opsonize cell debris for phagocytic removal, which is a normal physiological process during turnover. Furthermore, in the development of AMD, complement positively contributes to disease processes by tagging debris for phagocytic removal, which in turn contributes to the removal of damaged material and an anti-inflammatory microglial phenotype [142].

Recently, the complement system has become a new therapeutic target for AMD, particularly its advanced forms. While complement inhibitors have up to now failed to treat AMD successfully, emerging gene therapy may offer new opportunities to treat AMD in the future [143, 144]. Associations have been found between polymorphisms in the complement pathway and mtDNA damage of RPE in AMD. In cell culture, a more pronounced deterioration in mitochondrial function and increased inflammatory markers were observed in complement factor H (CFH) high-risk cells from each phase of the AMD state [145]. These results provide evidence for a newly recognized link between mitochondrial dysfunction and CFH that could contribute to RPE damage in AMD patients harboring the CFH high-risk genotype [146].

**Fig. 9:**
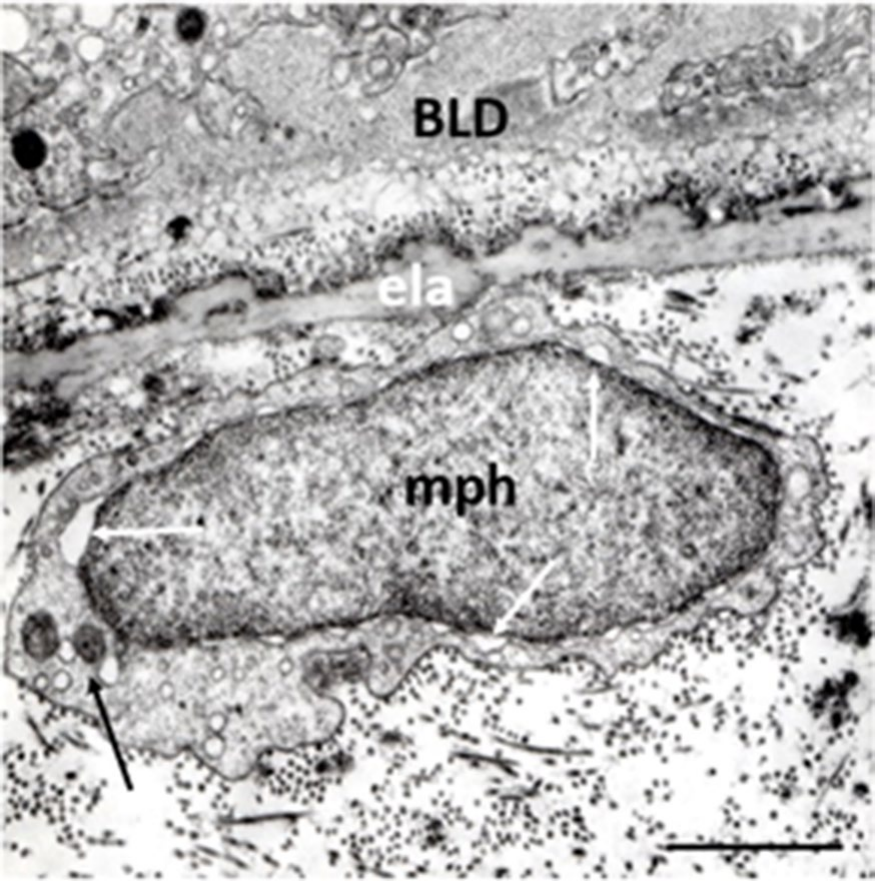
Macrophage in early AMD. Oval-shape macrophage in the intercapillary zone next to the elastic layer of the BM. The narrow cytoplasm is poor in cytoplasmic processes, it contains two round-shape mitochondria with confluent electrodense cristae and several endosomes. One of the mitochondria forms contact site with a lysosome (black arrow). The nuclear membrane is dilated nearly all round the nucleus (white arrows). Seventy-two years; male; ×22,000; bar: 1 μm

Histopathologic studies in early AMD have shown macrophages/microglia localized to pathologic areas of Bruch’s membrane where they insert processes into Bruch’s membrane deposits, presumably to scavenge debris, and these alterations were considered to be immune-mediated local inflammation [147]. Further studies showed that accumulation and extracellular deposition of metabolic by-products is associated with polarization of resident choroidal macrophages and recruitment of macrophages to Bruch’s membrane [148]. In an excellent whole-mount immunohistochemical study on human donor eyes, significantly more macrophages were found in the submacular choroid in all AMD groups as compared to aged control eyes [149]. Macrophages in eyes with early AMD were observed in the outer collagenous layer, next to the elastic layer of Bruch’s membrane (Fig. 9).

Emerging evidence suggests that the presence of activated immune cells in the subretinal space is associated with the up-regulation of purinergic signaling pathways [150]. Purines and their derivatives, mainly adenosine, and ATP, are the key molecules controlling intracellular energy homeostasis and nucleotide synthesis in the healthy CNS. Purines act as endogenous ligands that bind to and activate purinoceptors and subsequent purinergic signaling [141]. However, in neurodegenerative diseases, purinergic signaling becomes dysregulated, and significant amounts of various purine nucleotides are released into the extracellular matrix [151]. These purine nucleotides are also potent extracellular signaling molecules that can induce purinergic signaling pathways via activation of purinoceptors P1 and P2 that elicit a variety of pathophysiological pathways in the retina contributing to three types of blinding diseases including AMD, diabetic retinopathy, and glaucoma [152]. Recent studies identified a special sub-type of macrophages, capillary-associated microglia, which through P2Y purinoceptors regulate neurovascular structure and function [153]. P2Y purinoceptor agonists may indirectly inhibit microglia activity, thus they may have therapeutic importance in neuroinflammatory-neurodegenerative disorders of the retina. [154]. The P2X7 receptor is primarily known in microglial cells for its immune signaling and NLRP3 inflammasome activation. The P2X7 receptor modulates the clearance of extracellular and intracellular debris by microglial cells through modifications of lysosomal function, phagocytosis, and autophagy and mediates lysosomal damage that can activate the NLRP3 inflammasome [155]. Loss of the P2X7 purinoceptor function in mice induces retinal changes representing characteristics of early AMD [156].

Macrophages may also contribute to neovascularization. Advanced glycation end-product receptors are present on endothelial cells and monocytes, and they participate in the activation of these cells in inflammatory conditions. The ability of macrophages to promote vascular growth has been associated with the secretion and local delivery of proangiogenic factors, such as VEGF-A and proteases. Several studies have also revealed that physical contact of macrophages with growing blood vessels coordinates the vascular fusion of emerging sprouts. However, the interactions between macrophages and endothelial cells appear to be bidirectional, such that activated endothelial cells also support the expansion and differentiation of pro-angiogenic macrophages from myeloid progenitors [157, 158].

Mitochondria, in addition to their conventional role of meeting the cell’s energy requirements, also actively regulate innate immune responses. Several components of mitochondria, when released or exposed in response to damage, can be directly recognized by receptors of the innate immune system and trigger an immune response [159]. Furthermore, numerous innate immune responses are subject to mitochondrial regulation or require mitochondrial components. Finally, the metabolic state of the mitochondria within innate immune cells and mitochondrial metabolites modulates immune responses to stimuli [160]. MCSs are also involved in mitochondrial contribution to innate immunity and inflammation. There is an interplay between mitochondria and other cellular processes, such as autophagy, in controlling mitochondrial homeostasis as well as in the regulation of innate immunity and inflammatory responses [161, 162]. Moreover, MCSs have been observed in cells of the innate immune system, especially in macrophages [163]. Furthermore, microglia become progressively activated and seemingly dysfunctional with age, and genetic studies have linked these cells to the pathogenesis of a growing number of neurodegenerative diseases. With aging in mice and human brains, these “lipid-droplet-accumulating microglia” were defective in phagocytosis, producing high levels of ROS and secreting proinflammatory cytokines [164]. Phagocytic function of monocytes was also reduced in AMD, irrespective of the stage of the disease; as such, assessing peripheral monocyte phagocytic function can provide further insights into the etiology of this disease [165].

### Ganglion cell alterations

Choroidal ganglion cells (CGCs), with their neurons, form an intrinsic choroidal plexus. They are multipolar neurons of 20–40 μm in size and form an interconnected plexus that makes extensive contacts with both choroidal blood vessels as well as with non-vascular choroidal smooth muscle cells. They appear to be neither cholinergic nor adrenergic neurons but were stained with antibodies against neuronal nitric oxide synthase (nNOS) and vasoactive intestinal peptide (VIP) [166]. The CGCs were more numerous in the central choroid, specifically in a circumferential area corresponding to the entrance of the short posterior ciliary arteries and in the vicinity of the submacular area [167]*.*

The CGCs receive parasympathetic, sympathetic, and trigeminal sensory nerve fibers that regulate choroidal blood flow. The parasympathetic innervation has been shown to vasodilate and increase choroidal blood flow, while the sympathetic innervation has been shown to vasoconstrict and decrease choroidal blood flow. This is suggesting a significant age-related decline in VIP-positive parasympathetic nerve fibers and in vessel diameter accompanied by decreased macular choroidal blood flow in the submacular choroid of disease-free human donor eyes, suggesting that a decline in the neural control of choroidal blood flow and vessel diameter may explain the age-related decline in macular choroidal blood flow and its adaptive control observed clinically with aging [168].

The sensory innervation has been shown to both convey pain (light-induced pain called photophobia) and thermal information centrally and act locally to vasodilate and increase choroidal blood flow by releasing neuropeptides both proinflammatory substance P (SP) and calcitonin gen-related peptide (CGRP) and anti-inflammatory somatostatin [169]. Neuropeptides and their receptors are expressed widely in mammalian retinas, where they exert neuromodulator functions; and in multiple models of retinal diseases, different peptidergic substances play neuroprotective actions [170]. Neuropeptides, in addition to regulation of blood-flow and their neuroprotective role, contribute to innate immune functions. Substance P mediates interactions between neurons and immune cells, modulating immune cell proliferation rates and cytokine production [171]. Substance P is able to activate several immune cells, such as macrophages, CD4+ and CD8+ T lymphocytes, NK cells, and mast cells [172]. Furthermore, sensory neurons directly promote angiogenesis via SP signaling in response to inflammation [173]. Human monocytes and macrophages express SP and its receptor, supporting the notion that SP is biologically involved in regulating the functions of these cells in an autocrine fashion [174]. Several novel concepts have emerged recently, suggesting that the modulation of the neuropeptide system may provide an entirely new set of pharmacological approaches. Endothelial transient receptor potential (TRP) channels represent potential therapeutic targets in multiple disorders featured by abnormal vascularization, including ischemic disorders, neurodegeneration, and retinal degeneration [175].

**Fig. 10:**
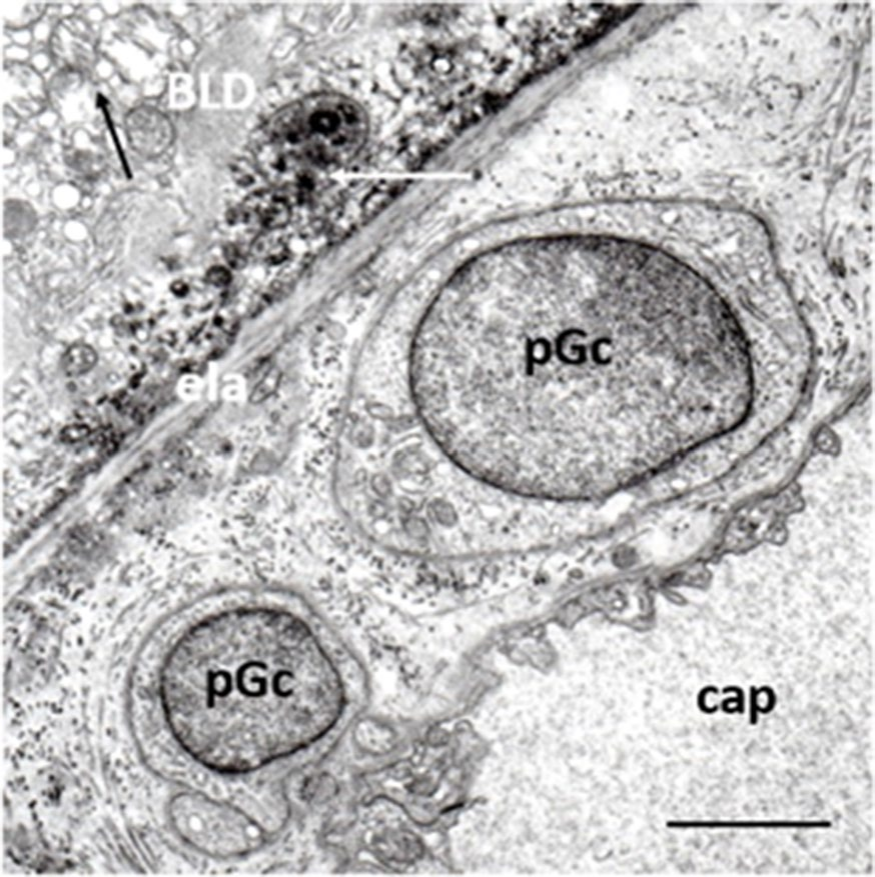
Ganglion cells (possible) in early AMD. Two round-shape cells located in the widened outer collagenous layer of the Bruch’s membrane (pGc). They have round nucleus, narrow cytoplasm which contain some mitochondria, endoplasmic reticula, and lysosomes. Thin basement membrane surrounds both cell bodies. Mitochondria with loss of cristae and matrix-density in the RPE (black arrow), basal linear deposits (white arrow), and BLD can also be seen. ela: elastic layer, cap: capillaries. Seventy-six years; female; ×19,000; bar: 1 μm

We observed previously unknown periendothelial cells in the choriocapillary layer next to the Bruch’s membrane in early AMD. Their electron microscopic features were starkly different from any other cells (macrophages and pericytes), and we suggest the term “proposed ganglion cell” (Fig. 10). Moreover, an intimate spatial relationship was found between their cytoplasmic processes and the choriocapillaris, suggesting that they may have intrinsic regulation of choriocapillary blood flow [176]. Further studies using neuro-specific markers are needed to identify these cells and to reveal their physiologic and pathophysiologic role in early AMD.

## Microbiota mitochondria intertwined relationship

### Microbial metabolites

In recent decades, significant attention has been focused on microbiota and on the influence of these several trillions of microorganisms on health and disease development. While symbiosis seems to be essential for health, dysbiosis (disorders of symbiosis) has been found to play a central role in different pathologies. Several studies on the mechanism of symbiosis and dysbiosis identified a series of microbial metabolites and compounds (fragments) that are responsible for both local and systemic effects on the metabolism, blood circulations, immune, and nerve systems. [177–179]. Furthermore, these studies also suggested that microbiota may be the common target of most epigenetic influences—including diet, stress, and physical activity [180, 181].

Recently, an International Scientific Consensus Panel proposed a common term “postbiotic” for microbial metabolites and compounds, unifying previously used terms (“killed probiotic,” “paraprobiotic,” “ghost probiotic,” “probiotic lysate,” “Tyndallized probiotics,” etc.), and defined postbiotic to be a “preparation of inanimate microorganisms and/or their components that confers a health benefit on the host”. Five mechanisms of postbiotic action have been postulated: (i) modulation of the resident microbiota; (ii) enhancement of epithelial barrier functions; (iii) modulation of local and systemic immune responses; (iv) modulation of systemic metabolic responses; (v) systemic signaling via the nervous system [182]. The Panel also recapitulated that dietary uptake of postbiotics may have similar or even better biological benefits as compared to their live counterparts. Furthermore, the delivery and safety of postbiotics are higher as compared to probiotics.

New research findings have highlighted that microbiota interacts with host cells, in particular by intermingling with the mitochondria and microbial metabolites released by microbes, which can directly influence mitochondrial metabolism and MQC. This microbiota—mitochondria cross-talk is intriguing because mitochondria share many common structural and functional features with the prokaryotic cells [183]. Another line of studies revealed that gut microbiota also has a significant influence on LDs in many cells. Accumulation of LDs is commonly observed in response to numerous host bacteria [184]. Microbiota-driven LDs biogenesis is a complexly regulated process that involves increased lipid uptake, new lipid synthesis, and innate immune receptors. Further studies demonstrated that pathogens are able to exploit LDs as an energy source, a replication site, and/or a mechanism of immune response evasion. Nevertheless, LDs can also act in favor of the host as part of the immune and inflammatory response to kill intracellular pathogens and regulate metabolic adaptation to infection [85].

Several studies also reported a correlation between microbiota quality, diversity, and mitochondrial function. Laboratory and clinical research suggested that the well-known mitochondrial disorder responsible for different pathological states may come from an imbalance of the microbial regulation [185–187]. Microbiota–mitochondria disorders have been described in gastrointestinal diseases, such as in new-onset Crohn’s disease [188], in chronic intestinal inflammation and cancer [69], in neuropsychiatric diseases like autism [189], Parkinson’s disease [190], Alzheimer’s disease [179], post-traumatic stress disorder [191], depression [192], chronic fatigue syndrome [193], in metabolic diseases such as obesity and type 2 diabetes [194], in chronic Marijuana users [195], in exercise [196], in aging [197], and recently in COVID-19 [198]. Here below we discuss influences and potential benefits of some microbial metabolites and compounds on AMD.

#### Microbial metabolites of fibers

Short-chain fatty acids (SCFAs), such as butyrate, acetate, and succinate, are the main metabolites produced in the colon by bacterial fermentation of dietary fibers and undigestible starch (“resistant starch”). Through binding to G protein-coupled receptors (GPCRs), such as free fatty acid receptor 2 and 3, SCFA have beneficial effects on mitochondrial activity and in neuro-immunoendocrine regulation in heath and disease. First of all, SCFAs influence intestinal mucosal epithelial barrier integrity and function, and, through systemic circulation, regulate liver metabolic function, insulin secretion by β-pancreatic cells, and whole-body energy homeostasis, as well as systemic immune functions and blood-brain barrier integrity by regulating the expression of tight junction proteins. Through interaction with enteroendocrine cells, SCFAs promote indirect signaling to the brain via the systemic circulation or vagal pathways by inducing the secretion of gut hormones such as glucagon-like peptide 1 (GLP1) and peptide YY (PYY), as well as γ-aminobutyric acid (GABA), and serotonin (5-HT). Finally, SCFAs also influence neuroinflammation by affecting glial cell morphology, continuously modulating microglia maturation, and also functioning to modulate the levels of neurotrophic factors, increasing neurogenesis, and improving neuronal homeostasis and function—known as gut microbiota-brain axis [187, 199].

Significantly altered SCFA levels were found in diseases associated with mitochondrial disorders such as autism spectrum disorder, affective disorders, multiple sclerosis, and age-related neurodegenerative diseases [200]. Acetate, one of the bacteria-derived SCFA molecules, was found to modulate microglial phagocytosis and disease progression in neurodegeneration [201]. Butyrate in cell lines from boys with autism positively modulated mitochondrial functions, including enhancing oxidative phosphorylation and β-oxidation in physiological stress and/or mitochondrial dysfunction. It has been proposed as a neuroprotectant metabolite that can help rescue energy metabolism during disease states [202]. Recent studies have shown that activation of the PGC1α signaling axis could be one of the molecular mechanisms underlying the beneficial effects of butyrate treatment in improving mitochondrial bioenergetics in NSC34-G93A cells. PGC1α is a master regulator of mitochondrial biogenesis [203].

#### Microbial metabolites of polyphenols

Polyphenols are a large family of natural compounds characterized by multiple phenol units. Recent studies on their interaction with gut microbiota and on microbial metabolites of polyphenols have opened up a new line of research and potentially new targets for application [204, 205]. Anthocyanins, common polyphenols in blueberries, have well-known antioxidant and anti-inflammatory properties and are widely used to attenuate visual disorders as anthocyanosides promote rhodopsin synthesis and regeneration, increasing retinal sensitivity to light intensity, improving visual acuity and dark adaptation—as well as the blood supply to the retina [206]. Anthocyanins showed retinoprotective effects by increasing the antioxidant defense mechanisms, suppressing lipid peroxidation and proinflammatory cytokines, and inhibiting apoptosis in a pigmented rabbit model of visible-light-induced retinal degeneration [207]. In vitro, anthocyanins attenuate light-induced photoreceptor cell damage [208]. Furthermore, the video display terminal (VDT) load-induced reduction in critical fusion frequency (CFF) and subjective asthenopia (eyestrain) symptoms were alleviated by anthocyanin supplementation in healthy humans [209]. In VDT workers, symptoms of dry eyes—such as foreign body sensation, eye fatigue, ocular pain, and eye heaviness—were also mitigated by anthocyanins [210]. Clinical studies showed that the impairment of cell clearance, redox homeostasis, anti-inflammatory, and antiangiogenic activity of the RPE may be attenuated by anthocyanin-containing phytochemicals and may have the potential to improve or halt the progression of AMD [211, 212].

The latest research findings revealed an interaction of polyphenols with both microbiota and mitochondria. Studies in colitis models indicate that polyphenols influenced microbiota composition, decreased opportunistic pathogenic or proinflammatory microbes, and increased probiotics such as Lactobacillus and Bifidobacterium, counteracting dysbiosis. Moreover, polyphenols also change microbial functions, such as increasing butyrate formation, and polyphenol metabolites produced by the gut microbiota appear to protect gut barrier integrity, interact with the immunometabolism, and mitigate inflammatory conditions in cells and animal models [213–215]. Antioxidant properties and phosphodiesterase inhibition by anthocyanins promote both mitochondrial function and density, which could be novel targets for the dietary management of obesity and its complications [216].

Urolithins are metabolites of gut bacteria-derived from dietary complex polyphenols such as pomegranates, berries, and nuts. Urolithin A (UA), one of the most studied microbial metabolites of polyphenols, enhances cellular health by increasing mitophagy and mitochondrial function and by reducing excessive inflammation. Several preclinical studies have shown its protective effects in aging and age-related diseases affecting muscle, brain, joints, and other organs. In humans, the benefits of UA supplementation in the muscle are supported by recent clinical trials in elderly people [217]. UA stimulated mitophagy and improved muscle function in older animals and induced mitochondrial gene expression in older humans. Benefits of long-term UA intake were observed in a randomized, double-blind, placebo-controlled clinical trial in adults aged 65 to 90 years. UA significantly improved muscle endurance and plasma biomarkers as acylcarnitines, ceramides, and CRP were decreased by UA, compared with a placebo [218, 219].

The therapeutic potential of UA for Alzheimer’s and Parkinson’s diseases has also been observed. Cell survival and mitochondrial length was significantly increased, and protein levels of mitochondrial fusion, synaptic and mitophagy genes increased, while mitochondrial fragmentation was reduced in treated mutant APP-HT22 cells. Furthermore, UA showed strong protective effects against mutant APP and Aβ-induced mitochondrial and synaptic toxicities in Alzheimer’s disease [220, 221]. In mouse models of Parkinson’s disease, UA reduced the loss of dopaminergic neurons and ameliorated behavioral deficits and neuroinflammation. The mechanism may be related to its inhibition of NLRP3 inflammasome activation via promoting mitophagy in microglia [222].

UA elicits enhancement of 1,25D-dependent mRNA encoding tryptophan hydroxylase-2 (TPH2), the serotonergic neuron-expressed initial enzyme in tryptophan metabolism to serotonin in cell culture [223]. In recent years, serotonin has emerged as a key neurotransmitter in the microbiota-gut–brain axis because it largely contributes to both gut and brain physiology, behavior, and cognitive function included [224]. AMD patients with higher levels of anxiety show a decrease in their quality of life, which depends on their visual functions [225]. These preliminary observations may open up a new approach for attenuating neuropsychiatric symptoms associated with AMD.

#### Microbial metabolites of bile acids

Primary bile acids (cholic acid and chenodeoxycholic acid) are synthesized in the liver from cholesterol, conjugated with glycine or taurine, and released into the intestine where it emulsifies and solubilizes food lipids for digestion. Gut microbiota secretes bile salt hydrolases and bile salt 7α-dehydroxylase which convert primary bile acids to the secondary bile acids—principally deoxycholic acid and ursodeoxycholic acid (UDCA)—that conjugate with taurine as tauroursodeoxycholic acid (TUDCA). Secondary bile acids regulate many physiological processes, including the lipid, carbohydrate, and energy metabolism of the host, but also shape the composition and function of the intestinal microbiota [226, 227].

Secondary bile acids UDCA and TUDCA are ligands for host cell nuclear receptors, farnesoid X receptor (FXR), G protein-coupled bile acid receptor 1 (TGR5), and vitamin D receptors (VDR) and have long been known to have antioxidant, antiapoptotic, and anti-inflammatory properties. Recent studies have demonstrated their benefits in neurological, neurodegenerative, and neuropsychiatric disorders, proposing bile acids as potential alternative therapeutic approaches for patients suffering from these disorders [87, 228]. The mechanisms underlying the protective activity have been mainly attributed to the alleviation of ER stress and stabilization of the unfolded protein response as a chemical chaperone [229].

Bear bile containing mainly UDCA and TUDCA has been used in Traditional Chinese Medicine for thousands of years due to its therapeutic potential. UDCA and TUDCA are approved in several countries, EU and USA included, for the treatment of certain cholestatic diseases. However, a growing body of research suggests their therapeutic benefits in a wide variety of non-liver diseases [230]. Earlier studies revealed the effects of UDCA and TUDCA on mitochondrial membranes and activity. UDCA reduced the mitochondrial membrane permeability transition in isolated mitochondria, and it was associated with significant prevention of mitochondrial membrane alterations [231]. Further study indicated that the neuroprotective role of TUDCA in the cell model is mediated by up-regulation of mitophagy. Thus, TUDCA prevents neurodegeneration as a modulator of mitochondrial activity and turnover [232]. TUDCA also significantly enhanced expression levels of mitochondrial biogenesis-related proteins and mitochondrial antioxidant responses in neural stem cells [233, 234]. In lipid oxidation processes induced by a Fenton reaction, TUDCA reveals substantial antioxidant activity [235].

Several experimental and clinical studies have described the benefits of secondary bile acids in retinal degeneration of various origins. TUDCA suppressed apoptosis and preserved the function and morphology of photoreceptors in two mice models of retinal degeneration [236]. It binds specifically to the light-activated rhodopsin and enhances phagocytosis of POS membranes by RPE [237, 238]. TUDCA can protect RPE cells against oxidative damage, ER stress, and inflammation [239]. TUDCA also showed immunomodulatory potential affecting the microglial phenotype in vivo and in vitro toward the anti-inflammatory [240, 241]. In vitro, TUDCA promoted RPE cell integrity and diminished VEGF-induced choroidal endothelial cell migration and tube formation [242], as well as protecting RPE tight junctions and inhibiting choroidal neovascularization in an in vitro model of neovascular AMD [243]. Systemic administration of UDCA and TUDCA significantly suppressed rat models of laser-induced choroidal neovascularization, and the VEGF levels in the retina were lower in the treated group as compared to controls, which might be associated with anti-inflammatory action [244]. Furthermore, systemic administration of UDCA and TUDCA also preserved photoreceptors after retinal detachment and upregulated antiapoptotic, antioxidant, and anti-inflammatory genes, suggesting that their oral administration may be a potential neuroprotective adjuvant therapy in retinal detachment and other retinal degenerative diseases like AMD [245, 246].

### Microbial compounds

In addition to microbial metabolites released by live microorganisms, microbial compounds derived from killed bacteria exert biological influences on the host organism. Endotoxin (LPS), peptidoglycan (PG), and pathogen-associated molecular pattern (PAMP) can reach the brain and eye through circulation, where they stimulate toll-like receptors (TLRs) of microglia, increasing the production of pro-inflammatory cytokines—such as IL-1β, IL-6, and TNF-α—resulting in neuroinflammation [247, 248]. Surprisingly, studies also revealed gene expression for TLRs as potential targets of microbial compounds in human RPE cells [249]. The activation of TLR2-3-4 induces pro-inflammatory responses in the RPE that may contribute to low-grade inflammation, modifying RPE functions and contributing to RPE degeneration [250]. In contrast, TLR9 recognizes viral or bacterial DNA with unmethylated CpG motifs, which decreases inflammation and enhances phagocytosis in RPE cells [251]. The protective effect of CpG oligodinucleotide in the mitigation of apoptosis is dependent on the reduction of ROS [252]. PCR analysis also showed gene expression of TLR1-6 and 9 in human choroidal endothelial cells [253]. These findings suggest that in addition to immune cells, RPE and endothelial cells may also contribute to microbiota-induced inflammatory responses of the retina.

Recent studies have shown that certain microbial compounds may have benefits on immune disorders evoked by dysbiosis in neurodegenerative diseases. Microbial compounds from heat-killed probiotic Lactobacillus fermentum increased mitochondrial activity and lipid metabolism, decreased the LDs content, and at the same time, improved age-related physiological features and extended the lifespan of Caenorhabditis elegans [254]. Treatment containing lysate of probiotics decreased amyloid beta accumulation and the progression of symptoms in transgenic mice model of Alzheimer’s disease. Additionally, exercise enhanced these benefits [255]. In in vitro studies, it has also been shown that heat-killed Lactobacillus plantarum has neuroprotective effects [256]. Moreover, in vitro and animal studies have demonstrated that intake of heat-killed probiotic Lactobacillus paracasei suppressed inflammation and photoreceptor degeneration in a murine model of light-induced retinopathy. These results suggest that it may have a preventive effect against retinal degenerative diseases [257–259].

## Conclusions

**Fig. 11:**
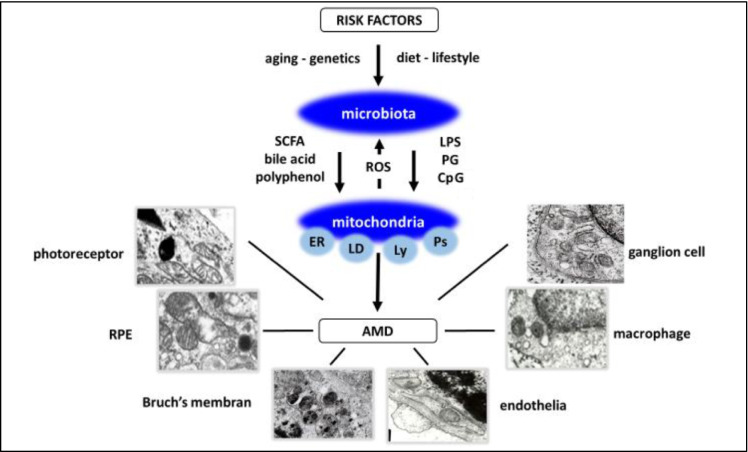
Microbiota–mitochondria disorders in early AMD. A proposed concept. Risk factors of AMD, aging, genetics, diet, and lifestyle are transduced by microbiota. Microbiota is established in early life and continuously reshaped by environmental influences throughout life. Microbial metabolites and compounds (‘healthy’ or ‘unhealthy’) released mainly in the gut regulate epithelial barrier and local mechanism, and through systemic circulation, gut-liver, and gut-brain axis they affect other organs, eye included. Mitochondria, the powerhouse of cells in addition to energy supply, through MCSs, an organelle-network of mitochondria, endoplasmic reticulum (ER), lipid droplets (LD), lysosomes (Ly), and peroxisomes (Ps) regulate main cellular functions including photoreceptor turnover, autophagy, and MQC. Mitochondrial ROS also influences microbial composition and function. Mitochondria are the organelle target of microbiota metabolites and compounds. SCFA, polyphenols, bile acids, and unmethylated CpG containing oligonucleotides improve metabolism and have anti-inflammatory, while endotoxins (LPS and PG) have proinflammatory effects. Microbiota-mitochondria disorders affect local and systemic metabolism, circulation, and neuro-immune surveillance and result in alterations of photoreceptors, RPE cells, Bruch’s membrane, endothelium, microglia, and ganglion cells of the choriocapillaris in early AMD

In industrialized countries, with an increase in the senior population, age-related neurodegenerative diseases have progressively become greater medical and socioeconomic burdens. These diseases have well-known common risk factors and common pathologic characteristics, such as the accumulation of metabolic by-products and chronic low-grade inflammation. Although enormous effort has been devoted to understanding the pathogenic mechanisms of neurodegenerative diseases, interventions to prevent or delay disease progression are largely ineffective, and the development of new strategies targeting these pathological features has long remained an urgent demand. In the present paper, we have outlined recent experimental and clinical studies supporting a new concept on the role of microbiota–mitochondria disorders binding risk factors and the manifestations of AMD (Fig. 11). We integrated our own observations into this concept to strengthen new strategies for the diagnosis and treatment of early AMD, and hopefully for other neurodegenerative diseases that share several common features of this blinding eye disease.

## Supplementary Information

ESM 1 (DOCX 55 kb)
