# Supplementary material for: Microbiota mitochondria disorders as hubs for early age-related macular degeneration
Source: GeroScience. 2022 Aug 18;44(6):2623–53. doi: 10.1007/s11357-022-00620-5 (PMC9385247; doi:10.1007/s11357-022-00620-5)
Supplement: Supplementary file 1 — (DOCX 55 kb) [file 11357_2022_620_MOESM1_ESM.docx]

**Supplementary information**

Material and Methods

Selection of material

Sixty-eight human eyes were involved in these histopathologic studies (aged 8 to 87 years, mean age 71.9 years). Thirty-four of them had early AMD (20 female and 14 male), and 34 eyes were used as age-and sex matched normal controls. The selection of early AMD was based on presence of drusen <125 μm and no drusen for control observed by light microscopy [3, 275]. All these human eyes were surgically removed because of malignant tumor or severe ocular trauma, neither of which affected the posterior pole of the eyeball. The study was conducted in keeping with the tenets of the Declaration of Helsinki. Written consent was obtained from all patients.

Transmission electron microscopy:

Small pieces of the retina and choroid were dissected at the posterior pole immediately (<2 minutes) after the removal of the eyeball and fixed at 4°C in 2% buffered glutaraldehyde for 2 h and postfixed in 2% osmium tetroxide for another 2 h. The postfixation with osmium tetroxide demonstrates membrane lipids and lipid peroxides effectively without damage to membrane structures [276]. The specimens were dehydrated, embedded in araldite, sectioned with a Reichert Ultramicrotome, contrasted with lead citrate and uranyl acetate, and studied

with a Zeiss 109 Electron Microscope.

Light microscopy:

The rest of the eyeballs were fixed in 10% buffered formaldehyde for 48 h. then dissected and one half of each was dehydrated, embedded in paraffin, and 6 µm thick sections were stained with hematoxylin-eosin and Periodic acid Schiff (PAS)-alcian-blue staining to detect polysaccharide containing structures.

Histochemistry - Polarization microscopy:

For the study of lipids, the other halves of the formaldehyde fixed eyeballs were washed with tap water and without dehydration were embedded in gelatin. Cryostat sections, 10 µm thick, were mounted in gum Arabic to suppress the birefringence of all structures except lipids. For lipid extraction, a methanol-chloroform 1:3 mixture was used for 24 h.

For the studies on proteoglycan Romhanyi’s Aldehyde-Bisulfite-Toluidine Blue (ABT) staining reactions were used [277]. This technique allows the polarization microscopic studies of neighboring OH groups based on the aldehyde-bisulfite addition reaction followed by toluidine blue staining at pH1.0. During the procedures, OH groups are transformed into dialdehydes by periodic acid and then transformed into negatively charged groups by the bisulfite addition reaction. In this way, they are rendered capable of binding toluidine blue at low pH, which results in basophilia and anisotropy indicating the linear order of the OH groups in the reacting macromolecules. In detail, the deparaffined sections were first treated with 0.5% periodic acid for 30 minutes, then with a saturated solution of sodium bisulfite, also for 30 minutes. After a short rinsing with water, the slices were stained for 5 minutes with toluidine blue at pH 1.0 (0.1% toluidine blue in 0.1 normal HCl). The controls were stained with toluidine blue at pH 4.5 (0.1% toluidine blue in McIlvain buffer). After staining, the dye solution was blotted off with filter paper and then 1% potassium ferricyanide solution was dropped onto the slides. This resulted in stabilization of the dye binding in the oriented state in which the dye had been bound by the structures originally and in maintaining its optical effects: the metachromatic basophilia and anisotropy. Without being rinsed in water the slices were mounted in gum Arabic containing 1% potassium ferricyanide. This gum Arabic layer was allowed to dry for 2–3 days.

For the study on collagen fibrils the deparaffined sections were mounted in 50% phenol-containing Canada balsam. Addition of phenol specifically inverses and increases the anisotropy of collagen fibers but not the other protein fibrils, like amyloid.

The samples prepared for polarization microscopy were studied with Leitz-Orthoplan-Pol microscope (Oberkochen, Germany). The light retardation (anisotropy) was measured with Brace-Kohler rotary compensator in 580 nm light. Five serial sections for every specimen were used, and the light retardation was measured in 10 different areas of each section. Thus, every data point represented a mean value of 50 measurements.

Statistical analysis

Polarization microscopic pictures of the same resolution for quantitative evaluation of collagen, lipid and proteoglycan content of Bruch’s membrane were used for computer assisted morphometric analysis. Data were expressed as mean±SD and analyzed using Statistica 6.0 (Statsoft, Inc. Tulsa, OK, USA), the significance level was p<0.05 in all statistical analyses. Regression analysis was performed to evaluate the correlation between age and different electron microscopic morphological alterations in aged and AMD group.
